# *Helicobacter* species in cancers of the gallbladder and extrahepatic biliary tract

**DOI:** 10.1038/sj.bjc.6604780

**Published:** 2008-11-25

**Authors:** C de Martel, M Plummer, J Parsonnet, L-J van Doorn, S Franceschi

**Affiliations:** 1 International Agency for Research on Cancer, 150 cours Albert Thomas, 69372 Lyon cedex 08, France; 2 Department of Medicine, Stanford University School of Medicine, 300 Pasteur Drive, S-169, Mail Code 5107, Stanford, CA 94305-5107, USA; 3 DDL Diagnostic Laboratory, Fonteijnenburghlaan 7, 2275 CX Voorburg, The Netherlands

**Keywords:** *Helicobacter*, biliary tract neoplasms, gallbladder neoplasms, gallstones, cholecystitis

## Abstract

*Helicobacter* species have been found in human bile and biliary tract (BT) tissue and are suspected to cause BT diseases, including gallbladder and extrahepatic cancers, collectively referred to in this work as BT cancers. We conducted a literature review of the epidemiological evidence linking the presence of *Helicobacter* species in bile or BT biopsies to BT cancers and benign diseases. Reports showed great variability with respect to study methods. Nine studies of BT cancers were identified, all with 30 or fewer BT cancers; eight included cancer-free control subjects and used polymerase chain reaction (PCR) as a means of *Helicobacter* species detection. In four of these studies, *Helicobacter* species were detected in patients with BT cancer significantly more frequently than in controls, at least when controls without BT diseases were used. In two studies, no *Helicobacter* species were detected in either cases or controls. *Helicobacter* species were also often detected in benign BT diseases such as gallstone disease or chronic cholecystitis. As our current knowledge relies on a few small studies that showed substantial differences, larger studies and more standardised protocols for detecting DNA and antibodies against *Helicobacter* species are needed to investigate a potential association with BT cancer.

Gallbladder cancers (ICD-10 code C23) and extrahepatic biliary tract cancers (code C24, hereafter referred to collectively as biliary tract (BT) cancers) are relatively rare malignancies with wide variations in incidence rates worldwide (Figure 1). The highest BT cancer incidence rates are seen within Latin America (e.g., up to 9.3 per 100 000 for men and 25.3 per 100 000 for women in Chile), but the disease is also frequent in Northern India, Japan, Korea, and some Eastern European countries (Curado *et al*, 2007). Although gallbladder cancer is the most common BT cancer, its overall incidence, and its proportion of all BT cancer, varies substantially across regions and genders (e.g., 18.9% among men in Denmark and 93.6% among women in New Delhi, India, Figure 1) (Saika and Matsuda, 2007). It usually occurs in patients with a history of gallstone disease and chronic cholecystitis (Randi *et al*, 2006).

*Helicobacter pylori* (*H. pylori*) infection is a well-established cause of stomach cancer (Amieva and El-Omar, 2008). Since the discovery of *H. pylori* in 1982, 30 other *Helicobacter* species have been isolated from the stomach, intestinal tract, and liver of mammals and birds. A few species found in human bile and BT tissue biopsies (*Helicobacter bilis (H. bilis)*, *Helicobacter pullorum (H. pullorum)*, *Helicobacter hepaticus (H. hepaticus)*, *and H. pylori*) have been suspected to cause BT diseases. We aimed to review current information on *Helicobacter* species in BT cancer and benign BT diseases in humans and to help delineate future research needs on the topic.

## Materials and methods

We carried out several detailed searches of the database MEDLINE through PUBMED, using the following entry terms in the first search round: (‘Gallbladder Neoplasms’[Mesh] OR ‘Gallbladder’[Mesh] OR ‘Gallbladder Diseases’[Mesh] OR Gallbladder [Text Word]) AND (‘Helicobacter’[Mesh] OR ‘Helicobacter Infections’[Mesh] OR ‘Helicobacter hepaticus’[Mesh] OR ‘Helicobacter pylori’[Mesh] OR ‘Helicobacter’[Text Word]). We then repeated the search using ‘Biliary tract neoplasms’, ‘Cholangiocarcinoma’, ‘Cholelithiasis’, ‘Cholecystis’, and ‘Gallstone’ as entry terms instead of ‘Gallbladder neoplasms’. Other MEDLINE searches were subsequently carried out using ‘Helicobacter bilis’ and ‘Helicobacter pullorum’ as single entry terms. Regional databases for India, Southeast Asia, and Latin America (the Index Medicus for South-East Asia Region (IMSEAR), Latin American and Caribbean Centre on Health Sciences Information (LILACS) and Indian Medlars Center National Informatics Centre (INDMED) databases, respectively) were also searched. Finally, we reviewed the reference lists of all identified relevant studies. No restrictions on date or publication language were applied. After exclusion of case-series with fewer than five patients or biological specimens, we retained all relevant human studies on BT cancer and their possible or proven precursor lesions published up to January 2008.

For the sake of accuracy and comparability, only the following methods of direct *Helicobacter* species detection in bile, gallstones, or tissue biopsies were considered for the review: (1) amplification by polymerase chain reaction (PCR), (2) histology (histopathology, immunohistopathology), and (3) culture. For each of the studies selected in this review, the following was retrieved whenever available: location and year of data collection, histological diagnosis, age and gender of BT cancer cases, type of biological specimen, method of *Helicobacter* species detection, type of *Helicobacter* species identified, and selection criteria of control subjects, if present.

## Results

### *Helicobacter* species and BT cancers

Nine studies (Table 1) that investigated the presence of *Helicobacter* species in the bile or BT tissue biopsies of patients with BT cancers were identified (Roe *et al*, 1999; Fukuda *et al*, 2002; Matsukura *et al*, 2002; Bulajic *et al*, 2002a; Fallone *et al*, 2003; Murata *et al*, 2004; Pradhan and Dali, 2004; Kobayashi *et al*, 2005; Bohr *et al*, 2007). Eight studies used PCR as one of the means of *Helicobacter* species detection. The PCR primers varied across studies, but most used genus-specific primers (such as C97-C98) as a first-line test before undertaking PCR assays that targeted different *Helicobacter* species.

Variability among PCR study findings was substantial (between 0 and 82.8% of specimens tested positive for *Helicobacter* species) even when analysis was restricted to the same type of biological specimen (bile or tissue biopsies). Using species-specific primers, *H. bilis* was found in 35 out of 67 specimens (52.2%) from four different studies, whereas *H. hepaticus* was searched for in two studies, but only found in 4 out of 19 specimens (21.1%) in one study.

Two studies used histopathology staining to search for *Helicobacter* species in BT tissue biopsies. Although *Helicobacter*-like bacteria were detected in six out of seven BT cancers from a study in Nepal, no infections were detected in a German series of 20 BT cancers, using three different methods of staining and histoimmunochemistry. The German study also reported negative findings according to several PCR amplification methods, as well as culture, of fresh gallbladder tissue biopsies. Only one other study reported an attempt to cultivate these bacteria, but although tissue biopsies yielded positive PCR results, culture in microaerophilic conditions was unsuccessful (Table 1).

Eight studies had one or more control groups allowing some comparison with cancer cases (Table 2). Controls were subjects who underwent the same procedure as cases, and were diagnosed with benign BT diseases. In three studies, a group of controls without BT diseases was also included. The presence of *Helicobacter* species in bile or BT tissue biopsies was detected significantly more often in cases than in controls in four studies, at least when controls without BT diseases were used, but none were detected in cases in two other studies (Table 2).

### *Helicobacter* species and benign BT diseases

Our literature search identified 20 studies investigating the presence of *Helicobacter* species in the bile, gallstones, or BT tissue biopsies of patients with benign BT diseases (Arnaout *et al*, 1990; Figura *et al*, 1998; Fox *et al*, 1998; Monti *et al*, 1999; Rudi *et al*, 1999; Myung *et al*, 2000; Harada *et al*, 2001; Mendez-Sanchez *et al*, 2001; Monstein *et al*, 2002; Roosendaal *et al*, 2002; Bulajic *et al*, 2002b; Chen *et al*, 2003, 2007; Silva *et al*, 2003; Farshad *et al*, 2004; Abayli *et al*, 2005; Apostolov *et al*, 2005; Neri *et al*, 2005; Tiwari *et al*, 2006; Misra *et al*, 2007). Of these, 19 used PCR amplification methods (Table 3). The choice of primers varied across studies; some primers were based on genes coding for the 26kDa *H. pylori* protein, *UreA* or *UreB* enzymes, whereas others targeted 16S ribosomal RNA fragments, either common to all organisms of the *Helicobacter* genus, or specific to a particular species. Only in two studies, from Chile and Brazil, did the authors undertake sequencing to identify the detected species.

*Helicobacter* species were assessed by PCR in gallstones in five studies. The frequency of detection varied from 0 to 72%, and the species identified was believed to be *H. pylori* in all cases. In one study from Sweden, *H. pylori* was searched for and detected in the nucleus rather than in the envelope of the stones, suggesting an early presence of the bacteria in the process of gallstone formation (Monstein *et al*, 2002). Of 16 PCR studies on *Helicobacter* species in bile or BT tissue biopsies in benign BT diseases, the percentage of positive specimens varied from near 0% in five studies to close to 50% in six others, with three studies showing intermediate percentages. Two other studies reported a high prevalence: 29 out of 30 bile specimens of patients with hepatobiliary diseases were *Helicobacter* genus-positive in one study, as were 16 out of 22 gallbladder tissue biopsies of chronic cholecystitis patients in another.

Histological and/or immunohistological examination of tissue biopsies was undertaken in seven studies (including two histological series of metaplastic gallbladder sections) using various staining methods and antibodies. *Helicobacter*-like bacteria were seen in all but one study, and the percentage of positive specimens varied from 1 to 45%. In three studies, immunohistological staining using anti-*H. pylori* antibodies confirmed the histological findings. Attempts to culture the bacteria were reported in four studies, with three failing to grow any *Helicobacter* species from frozen specimens, despite some success with PCR or histology. In another study, however, 6 out of 77 (8%) fresh gallbladder tissue biopsies from patients with gallstones grew *Helicobacter*-like bacteria (Table 3).

## Discussion

We aimed to evaluate available evidence linking *Helicobacter* infection with BT cancer. It became clear that our current knowledge relies mainly on a few small studies that show substantial differences in methods and results. In four studies, mainly from Japan, the detection of *Helicobacter* species was significantly more frequent in bile or BT tissue biopsies of cancer patients compared with controls, at least when controls without BT diseases were used. Using species-specific primers, the *Helicobacter* species most consistently searched for and identified from bile or BT tissue biopsies of cancer patients was *H. bilis*. In two studies from Canada and Germany, however, no *Helicobacter* species were detected in BT cancers, despite the use in the German study of numerous detection techniques.

The presence of *Helicobacter* species, including *H. pylori*, was also often detected in benign BT diseases such as gallstone disease or chronic cholecystitis, which are recognised risk factors for the development of BT cancer. However, as with BT cancer, studies of benign BT diseases showed extreme variability in methods and findings.

Lower *Helicobacter* species prevalence was typically observed in western countries with low BT cancer incidence, and higher prevalence in countries with high BT cancer incidence, the best example being Japan. Whether regional variations in the prevalence of *Helicobacter* species in BT cancers are real or are a result of differences in the type and quality of detection methods used is unknown.

Most findings presented here derive from PCR-based studies, the comparability of which depends on the quality of the biological specimens (bile, stone, or tissue biopsies; fresh, fixed, or frozen), the strategy chosen for *Helicobacter* detection, and potential problems such as contamination and the presence of Taq polymerase inhibitors. The sensitivity and specificity of PCR are also directly dependent on the choice of primers. In early studies, primers targeting the genes encoding the 26kDa protein or the *UreA* or *UreB* proteins were often used. Although authors believed that *H. pylori* was identified, these findings may also be consistent with other *Helicobacter* species. For example, it has been shown that a gene coding for the 26kDa protein is present in at least eight other *Helicobacter* species (including *H. bilis* and *H. pullorum*) with high similarity to the gene in *H. pylori* (Lundstrom *et al*, 2001). Similarly, urease structural genes from *H. hepaticus* are highly homologous to *UreA* and *UreB* from *H. pylori* (Shen *et al*, 1998). Even PCR based on the conserved 16S rRNA genes may yield different results depending on the set of primers used (Moyaert *et al*, 2008).

It should be noted that the available sequence information from non-*H. pylori* species is still limited. Moreover, for *H. pylori*, it has been shown that the intraspecies sequence variability is substantial, which may hamper uniform detection by a single set of PCR primers (Kraft *et al*, 2006). Therefore, it is difficult to determine whether PCR primers can distinguish *Helicobacter* species, especially in patients from different geographic regions. The sensitivity of PCR is also inversely proportional to the length of the amplicon. This is particularly important in clinical materials with a high risk of DNA damage, such as formalin-fixed, paraffin-embedded tissue biopsies.

Taken together, for reliable *Helicobacter* species detection and distinction, it would be advisable, although seldom done so far, to use multiple PCR primer sets at somewhat reduced annealing temperatures to permit amplification of imperfectly matching sequences. Ultimately, amplicons obtained by such PCR should be sequenced to confirm the true identity of the *Helicobacter* species.

Means of detection other than PCR also have substantial drawbacks. *Helicobacter* species culture has been unsuccessful in the majority of studies of BT cancer or benign BT diseases. The use of frozen specimens, which are notoriously difficult to culture, may explain some of the negative findings (Solnick and Schauer, 2001). Histology has been considered the gold standard for the detection of *H. pylori* in the stomach for many years; however, few studies have assessed histological changes associated with *Helicobacter* infection in the gallbladder or BT tissue. In two studies from India and China, colonisation by *Helicobacter* was shown in the gallbladder epithelium, especially in the areas of gastric metaplasia (Chen *et al*, 2007; Misra *et al*, 2007), but it is not known whether this was merely a consequence of tissue damage. Serological studies have not been reviewed here, but cross-reactivity between the immune response to antigens from *H. pylori* and *H. bilis* has been reported (Ananieva *et al*, 2002; Pisani *et al*, 2008). Furthermore, it has been shown that the prevalence of serological and histological markers of *H. pylori* decreases in gastric cancer (Camorlinga-Ponce *et al*, 2008) and it is not known whether the same happens in BT cancer.

An implication of *Helicobacter* infection in BT cancer pathogenesis is nevertheless plausible. *Helicobacter* species that can survive in, or colonise, the bile ducts may induce the formation of gallstones both directly, through the urease activity of some of the species, or indirectly through a T-cell-dependent immune response (Belzer *et al*, 2006; Maurer *et al*, 2007). The colonisation of the mucosa by bacteria may also aggravate the chronic inflammatory state already caused by gallstones (Wistuba and Gazdar, 2004; Jergens *et al*, 2007). In animal studies, the sequence of events going from chronic inflammation to cancer has been directly linked to some *Helicobacter* species. For instance, *H. hepaticus* can cause chronic active infection of bile canaliculi that progresses to liver carcinoma in A/JCr laboratory strains of mice (Ward *et al*, 1994). Similarly, colon cancer in SMAD-3-deficient mice is enhanced by dual infection with *H. hepaticus* and *H. bilis* (Maggio-Price *et al*, 2006).

In conclusion, further development of PCR testing protocols is required, as well as a better characterisation of antigens suitable for histoimmunochemistry. As the BT is only accessible through invasive procedure or surgery, the choice of controls who can provide adequate specimens for case–control studies is limited. Larger epidemiological studies will only be possible by developing serological methods validated against direct detection of *Helicobacter* species in the gallbladder.

## Figures and Tables

**Figure 1:**
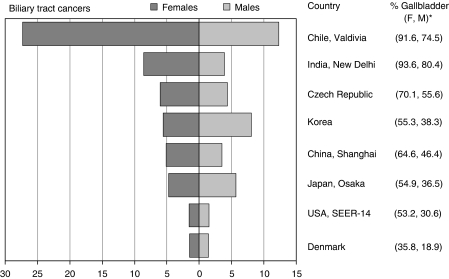
Age standardised incidences rates per 100 000 (world standard population) for biliary tract cancer, including gallbladder and extrahepatic biliary tract cancers (ICD-10, C23-C24) in selected areas of the world (1998–2002). ^*^Percentage of gallbladder cancers (code C23 only) on all biliary tract cancers in women (F) and men (M). Source: Curado *et al*, 2007.

**Table 1:** Human studies on *Helicobacter* (*H*) species and gallbladder cancers (GC) or extra-hepatic biliary tract cancers (EBC) by testing method

**Reference**	**Country**	**Cancer diagnosis**	**Biological specimen**	**Method**	**Organism**	**H-positive/ total cases**
**PCR**				**Primer**		
Roe *et al* (1999)	Korea	EBC	Bile	16S (H276f; H676r)	*H* genus	6/15
				*UreA* (HPU1; HPU2)	*UreA*-positive *H*	7/15
Bulajic *et al* (2002a)	Serbia	GC+EBC	Bile	16S (various), *UreA*	*H* genus	12/15
Fukuda *et al* (2002) a	Japan	GC+EBC	Bile, tissue biopsies	16S (C97–98)	*H* genus	10/19
				16S (species specific)	*H bilis*	6/19
				16S (species specific)	*H* *hepaticus*	4/19
				*UreA* (HPU1; HPU2)	*UreA*-positive *H*	1/19
Matsukura *et al* (2002)	Japan	EBC	Bile	16S (species specific)	*H bilis*	13/15
	Thailand	GC+EBC	Bile	16S (species specific)	*H bilis*	11/14
Fallone *et al* (2003)	Canada	EBC	Bile	16S (C97–98)	*H* genus	0/9
Murata *et al* (2004)	Japan	GC+EBC	Tissue biopsies	16S (species specific)	*H bilis*	4/14
Kobayashi *et al* (2005)	Japan	GC+EBC	Bile	16S (C97–98 or C05)	*H* genus	5/6
				16S (species specific)	*H* *bilis*	1/5
				16S (species specific)	*H hepaticus*	0/5
				*UreA*	*UreA*-positive *H*	2/6
Bohr *et al* (2007)	Germany	GC	Tissue biopsies	16S (C97–20; H3A-20)	*H* genus	0/20
						
**Histology**				**Stain, antibodies, etc.**		
Pradhan and Dali (2004)	Nepal	GC+ EBC	Tissue biopsies	Histopathology, Warthin-Starry	*H* genus	6/7
Bohr *et al* (2007)	Germany	GC	Tissue biopsies	Histopathology, Various staining	*H* genus	0/20
				Histoimmunochemistry	*H* genus	0/20
						
**Culture**				**Conditions**		
Roe *et al* (1999)	Korea	EBC	Bile	Fresh specimens, under microaerophilic and anaerobic conditions	*H* genus	0/15
Bohr *et al* (2007)	Germany	GC	Tissue biopsies	Fresh specimens, under microaerophilic conditions	*H* genus	0/5

a Five cases in this study were intra-hepatic bile duct cancers.

**Table 2:** *Helicobacter* (*H*) species detection in biliary tract (BT) cancer patients compared with controls

**Reference**	***H*-positive/total cases**	**Control diagnosis**	***H*-positive/total controls**
Roe *et al* (1999)	6/15	Benign BT diseases	3/11
Matsukura *et al* (2002)			
Japan	13/15	Benign BT diseases	8/16^a^
Thailand	11/14	Benign BT diseases	10/26^a^
			
Bulajic *et al* (2002a) b	12/15	No BT diseases	3/11^a^
		Benign BT diseases	37/63
			
Fukuda *et al* (2002) c	10/19	Benign BT diseases	3/19^a^
Fallone *et al* (2003)	0/9	Benign BT diseases	0/75
Murata *et al* (2004)	4/14	Benign BT diseases	2/16
Kobayashi *et al* (2005)	5/6	No BT diseases	2/21^a^
		Benign BT diseases	16/30
			
Bohr *et al* (2007)	0/20	No BT diseases	0/22
		Benign BT diseases	1/57^d^

a Fisher's exact test, *P*<0.05.

b Bulajic *et al* (2002a) also reported age- and sex-adjusted odds ratio (9.9; 95% confidence interval: 1.4–70.5).

c Five of the cases in this study were intrahepatic bile duct cancers.

d In the only positive control specimen, the organism was identified as *Helicobacter ganmani*.

**Table 3:** *Helicobacter (H)* species in patients with benign biliary tract diseases

**Reference**	**Country**	**Biological specimen**	**Method**	**Organism**	***H*-positive/ total cases**
**PCR**			**Primer**		
Figura *et al* (1998)	Italy	Bile	CagA (final product 298 bp)	CagA-positive *H*	1/30
Fox *et al* (1998)	Chile	Bile, tissue biopsies	16S (C97–98 or C97–05)	*H* genus	22/46
			Sequencing	*H* *bilis*^a^	7/8
			Sequencing	*H pullorum*	1/8
Monti *et al* (1999)	Argentina	Bile	Not specified (final product 296 bp)	*H* genus	2/26
Rudi *et al* (1999)	Germany	Bile	16S (various species specific)	*H* genus	0/73
Myung *et al* (2000)	Korea	Bile, tissue biopsies	*UreA* (final product 258 bp)	*H* genus	5/53
			26kDa	*H* genus	4/53
		Stone	*UreA* or 26kDa	*H* genus	7/53
			*UreA* or 26kDa	*H* genus	0/7
Harada *et al* (2001)	Japan	Bile, tissue biopsies	16S (C97–98)	*H* genus	2/53
			*UreA* (final product 258 bp)	*UreA*-positive *H*	1/53
Mendez-Sanchez *et al* (2001)	Mexico	Tissue biopsies	16S (C97–98)	*H* genus	1/95
Bulajic *et al* (2002b)	Serbia	Bile	*UreA* (final product 258 bp)	*H* genus	35/65
Monstein *et al* (2002)	Sweden	Stone	16S (species specific)	*H pylori*	11/20
Roosendaal *et al* (2002)	Netherlands	Bile	16S (various specific primers)	*H pylori*	1/21
Chen *et al* (2003)	New Zealand	Tissue biopsies	16S (C97–98)	*H* genus	46/85
			26kDa	*H* genus	31/46
			16S (species specific)	*H pylori*	22/25
			16S (C62-C12)	*H bilis*	0/85
Silva *et al* (2003)	Brazil	Bile, tissue biopsies	16S (C97–98)	*H* genus	18/46
			Sequencing	*H pylori*	18/18
Farshad *et al* (2004)	Iran	Stone	16S (species specific)	*H pylori*	6/33
		Bile	16S (species specific)	*H pylori*	4/33
Abayli *et al* (2005)	Turkey	Stone	16S (final product 349 bp)	*H* genus	7/77
Apostolov *et al* (2005)	Ukraine	Tissue biopsies	16S (C97–98)	*H* genus	16/22
Neri *et al* (2005)	Italy	Bile, tissue biopsies	16S (C95–98)	*H* genus	17/33
Tiwari *et al* (2006)	India	Bile	16S	*H* genus	29/30
			16S (species specific)	*H pylori*	10/29
			*UreA* (final product 411 bp)	*UreA*-positive *H*	10/29
			CagA	CagA-positive *H*	9/29
Chen *et al* (2007)	China	Tissue biopsies	*UreA* (final product 411 bp)	*UreA*-positive *H*	15/81
			*UreB* (final product 132 bp)	*UreB*-positive *H*	18/81
			*UreA* or *UreB*	Ure-positive *H*	22/81
Misra *et al* (2007)	India	Stone	16S (species specific)	*H pylori*	8/11
					
**Histology**			**Stain, antibodies, etc.**		
Arnaout *et al* (1990)	UK	Tissue biopsies	Histopathology, H&E (PAS), Warthin-Starry	*H* *pylori*	0/16^b^
Fox *et al* (1998)	Chile	Tissue biopsies	Histopathology, Warthin-Starry	*H* genus	2/18
Mendez-Sanchez *et al* (2001)	Mexico	Tissue biopsies	Histopathology, H&E, Giemsa	*H* genus	0/95
			Immuno-histopathology, polyclonal anti *H pylori*	*H* *pylori*	1/95
Abayli *et al* (2005)	Turkey	Tissue biopsies	Histopathology, H&E, gram, Warthin-Starry	*H* genus	18/77
Apostolov *et al* (2005)	Ukraine	Tissue biopsies	Immuno-histopathology, anti-CagA, anti-VacA, anti-*H pylori*	*H* *pylori*	13/16^c^
Chen *et al* (2007)	China	Tissue biopsies	Histopathology, Warthin-Starry	*H* genus	71/524
			Immuno-histopathology, polyclonal anti-*H pylori*	*H* *pylori*	37/71^d^
Misra *et al* (2007)	India	Tissue biopsies	Histopathology, H&E (PAS), Loeffler, Warthin Starry	*H* genus	50/111^b^
			Immuno-histopathology, polyclonal anti-*H pylori*	*H* *pylori*	50/111^b^
					
**Culture**			**Conditions**		
Fox *et al* (1998)	Chile	Bile, tissue biopsies	Frozen specimens, microaerophilic conditions	*H* genus	0/46
Harada *et al* (2001)	Japan	Bile	Frozen specimens, microaerophilic conditions	*H* genus	0/39
Silva *et al* (2003)	Brazil	Bile, tissue biopsies	Frozen specimens, microaerophilic conditions	*H* genus	0/46
Abayli *et al* (2005)	Turkey	Tissue biopsies	Fresh specimens, microaerophilic conditions	Oxydase- and ure-positive *H* genus	6/77

a *H* species identified in this study as *H rappini* was later reclassified as belonging to the *H bilis* species (Hanninen *et al*, 2005).

b Only specimens with gastric metaplasia were investigated.

c Only specimens *H* genus-positive by PCR were investigated.

d Only specimens *H* genus-positive by histology were investigated.
